# Effect of age, sex, breed and venipuncture site on platelet count and clumping in feline blood samples

**DOI:** 10.1177/1098612X241305919

**Published:** 2025-01-16

**Authors:** Sarah M Larkin, Matthew R Kornya

**Affiliations:** Department of Clinical Studies, Ontario Veterinary College, University of Guelph, Guelph, Ontario, Canada

**Keywords:** Hemostasis, phlebotomy, thrombocytopenia, thrombocytosis

## Abstract

**Objectives:**

To evaluate the associations between sex, age, breed and collection site on platelet count and platelet clumping in feline blood samples.

**Methods:**

Cats presenting to a primary care feline hospital from January 2016 to January 2017 were recruited. Any cat undergoing blood collection for a complete blood count was eligible. Cats were excluded if they were receiving clopidogrel or aspirin, had a disease known to affect platelet function, or if they required sedation for phlebotomy. All cats had their sex, age, site of venipuncture, platelet count, degree of platelet clumping and platelet morphology recorded.

**Results:**

In total, 649 cats were prospectively recruited. Of these, 579 (89%) cats had no clumping observed on blood smears. A significant association (*P* = 0.025) was found between sex and platelet count, with females having lower platelet counts. No significant association was found between sex and degree of platelet clumping (*P* = 0.323). Age did not have a statistically significant association with platelet clumping *(P* = 0.959); however, it did have a small significant (*P* = 0.003) positive correlation with platelet count. There was no significant effect of purebred status on platelet count (*P* = 0.457); however, the domestic group had a higher rate of platelet clumping (*P* = 0.009). No association was found between platelet count (*P* = 0.322) or degree of platelet clumping (*P* = 0.793) and collection site. When considering platelet clumping as a binary outcome, no association was found with sex (*P* = 0.292), age (*P* = 0.681), site of collection (*P* = 0.809) or breed (*P* = 0.264).

**Conclusions and relevance:**

The lack of effect of collection site/technique suggests that multiple sites of collection are valid when accurate platelet counts are important. The finding of lower platelet counts in younger and female cats may highlight the need to recognize age and sex when considering the management and monitoring of platelet counts and platelet disorders. Additional studies are needed to understand breed variation.

## Introduction

Platelets play an integral role in hemostasis and are of particular relevance in feline hematology.^1^ Assessing platelet count in cats and other species is critical given their involvement in a variety of conditions, including cardiomyopathies and thromboembolic disease.^2,3^ In cats, pseudothrombocytopenia (ie, falsely low platelet counts due to clumping) is well documented.^4–6^ Approximately 1.2–3.1% of cats presenting to veterinary hospitals are reported to be truly thrombocytopenic, although 47–71% have low platelet counts on automated analyzers, likely as a result of platelet clumping.^4,6,7^ When true thrombocytopenia is present, it may be linked to significant pathology such as viral infections or neoplasia.^6–8^ As such, low platelet counts must be confirmed with blood film analysis.^6^

In humans, differences have been noted in platelet count and function based on age, sex and ethnicity.^9,10^ Human platelet counts decrease with age in a non-linear fashion.^9,11^ Post-pubescent women have been documented to have increased platelet counts compared with men, as well as increased platelet reactivity in clopidogrel-treated patients.^3,9^ It has been postulated that this difference may have a hormonal basis, occurring due to higher body mass index in women, or changes in inflammation.^12,13^

In dogs, breed-associated differences in platelet aggregation, count and size have been noted.^14,15^ Cavalier King Charles Spaniels have a well-described benign macrothrombocytopenia, which follows an autosomal recessive mode of inheritance due to mutation in beta1-tubulin.^15–17^ A similar syndrome can be seen in Norfolk Terriers due to a different mutation in the same gene.^18^ In dogs, age, breed and sex are not associated with platelet count; however, weight is negatively correlated.^19^

There has been less investigation on the effects of signalment on platelet parameters in cats. Females are over-represented among thrombocytopenic cats, although breed and neuter status are similar between thrombocytopenic and control group cats.^8^ Similar to dogs, domestic and large cats have differences in gene sequences for the M loop of beta1-tubulin that may contribute to the variability in platelet size between cats and other mammals.^20^ While sex and age have been associated with feline lymphocyte counts, this has not been seen for platelet counts.^21^

Collection site and technique may also play a role in the degree of platelet clumping. Feline platelets likely aggregate in response to venipuncture.^5^ It has been postulated that increased platelet aggregation in cats may be due to the difficulties of venipuncture in this species; however, this has been disputed.^4,6^ If true, the site of collection may be related to this due to differing ease of collection. When comparing platelet counts in samples collected from a vascular access port or an 18 G jugular catheter with direct venipuncture, no differences were appreciated.^22,23^ When comparing platelet counts from blood taken via direct venipuncture through a 25 G vs 22 G needle, no clinically relevant difference was found.^24^

Minimal information is available on the effect of breed, sex and age on platelet count and clumping in cats. Given the individual variability observed in other species, the purpose of this study was to determine whether the venipuncture collection site, sex, age or breed are associated with the platelet count or degree of clumping in cats.

## Materials and methods

Cats presenting to a primary care feline hospital from January 2016 to January 2017 were prospectively recruited. Any cat undergoing blood collection for a complete blood count was eligible for enrollment. Cats were excluded if they were receiving clopidogrel or aspirin, if they had a disease known to affect platelet function, or if they required sedation for phlebotomy. All cats had their sex, age, site of venipuncture, platelet count, degree of platelet clumping and platelet morphology recorded.

Samples were collected from either the jugular or medial saphenous vein. Jugular blood samples were collected using a 23 G 1 inch needle and a 3 ml syringe with manual aspiration and transferred immediately to a 2 ml capacity K_2_EDTA vacutainer collection tube. Cats were in sternal or lateral recumbency during collection, which was not recorded. Medial saphenous samples were collected using a 23 G 0.75 inch butterfly catheter and collected directly into a 2 ml capacity K_2_EDTA vacutainer-style collection tube. All tubes were filled to their maximum recommended capacity. Cats were in lateral recumbency during collection. Isopropyl alcohol was used to prepare the collection sites. Tourniquets were not used. The choice of collection site and left vs right vein was at the phlebotomist’s discretion and was not based on any randomization or pre-study guidelines, nor was this recorded.

Samples were evaluated at a commercial reference laboratory with a XT-V series automated hematology analyzer (Sysmex). Quality control provided by the manufacturer was run on the analyzer three times a day during shift changes. A blood smear was made from each sample and reviewed in house by an author (MK) within 1 h of sample collection. Platelet clumps were investigated by examination of the feathered edge and the monolayer region, and the body of the smear was scanned at low power. The presence, number and size of platelet clumps were used to score samples as containing no, mild, moderate or marked platelet clumping using a standardized protocol (Table 1). The reference intervals used to categorize thrombocytopenia and thrombocytosis in this study were the general intervals used by the commercial laboratory generated as per Clinical & Laboratory Standards Institute guidelines.

**Table 1:** Criteria utilized to categorize cats based on the degree of platelet clumping

0	None/minimal	No visible clumps or one or two small clumps, platelet count subjectively normal
1	Mild	Multiple small clumps or one or two moderate clumps, platelet count subjectively normal
2	Moderate	Innumerable small, >2 moderate, or one large clump, platelet count subjectively low
3	Marked	None to rare free platelets, several large clumps

Small clump: fewer than five platelets; moderate clump: approximately 5–25 platelets; large clump: estimated more than 25 platelets

Due to small numbers of cats of most breeds, breed was dichotomized as ‘Domestic’ (including domestic shorthair, longhair and mediumhair) and ‘Purebred’ (all purebred cats). Due to small numbers of intact animals, sex was dichotomized as male or female, and the analysis was repeated with intact animals included or excluded. Cats with noted platelet clumping (mild, moderate or marked) were excluded from all analyses related to platelet count.

Statistical analysis was performed using commercial software (DataTab). Age and platelet count were treated as metric variables; sex, collection site and platelet morphology as nominal; and degree of platelet clumping as ordinal. Platelet count was assessed for normality using Shapiro–Wilk, Anderson–Darling and Kolmogorov–Smirnov tests.

The Dunn–Bonferroni test was used to assess the differences between the degrees of platelet clumping. A Mann–Whitney U test was used to determine whether there was a relationship between sex and platelet count and between sex and platelet clumping. Spearman correlation coefficients were used to determine whether age was associated with platelet count or platelet clumping. The Mann–Whitney U test was used to assess the relationship between breed and platelet count or degree of clumping, as well as between collection site and platelet count or degree of clumping.

Platelet clumping was also analyzed as a binary outcome (ie, clumped vs not) and a Mann–Whitney U test, Student’s *t* test and Kruskal–Wallis test were performed to assess its relationship to sex, age, site of collection and breed.

## Results

In total, 649 cats were recruited and met inclusion criteria. Of these, 322 were neutered males (N), 318 were spayed females (S), seven were intact males (M) and two were intact females (F). Cats ranged in age from 2 months to 23 years, with a mean, median and mode of 10.2, 11 and 12, respectively.

Platelet count did not follow a normal distribution. Of the 649 cats included, 579 cats (89%) had no platelet clumping observed on a blood smear (Table 2). The median platelet count was 301 × 10^9^/l (range 27–1185 × 10^9^/l). If cats with platelet clumping were excluded, the median platelet count was 314 × 10^9^/l (range 103–1185 × 10^9^/l). Excluding those with platelet clumping, thrombocytosis (platelet count above the reference interval: >641 × 10^9^/l) was present in 11 cats (1.9%) and thrombocytopenia (platelet count below the reference interval: <155 × 10^9^/l) was present in two cats (0.34%). Thrombocytopenia occurred in 70 cats (11%) when all cats were included regardless of the degree of platelet clumping. Thrombocytosis was positively associated with age (*P* = 0.009) but not sex (*P* = 0.765) or collection site (*P* = 0.730). Analysis of thrombocytopenic animals was not performed due to the small number of cats.

**Table 2:** The number of cats (frequency), median platelet count and platelet count interquartile range based on the observable degree of platelet clumping

Degree of platelet clumping	Number of cats (frequency)	Median platelet count (× 10^9^/l)	Interquartile range
0	579	340.45	+/– 148.5
1	15	124.37	+/– 28
2	27	107.15	+/– 38.5
3	28	131.57	+/– 59

A significant difference was found in platelet count between the four degrees of platelet clumping (*P* <0.001). A significant difference was present between groups 0 vs 1, 0 vs 2 and 0 vs 3 (*P* <0.001); no significant difference was seen between groups 2 vs 1, 3 vs 1 or 3 vs 2 (*P* = 0.807, *P* = 0.867 and *P* = 0.624, respectively).

A statistically significant association (*P* = 0.025) was present between sex and platelet count, with females having lower platelet counts (median females = 326.97 × 10^9^/l; median males = 354.27 × 10^9^/l). With intact animals excluded, a statistically significant difference was still present (*P* = 0.012), with the female group having lower values for platelet count (median of 327.19 × 10^9^/l vs 356.83 × 10^9^/l).

**Figure 1:**
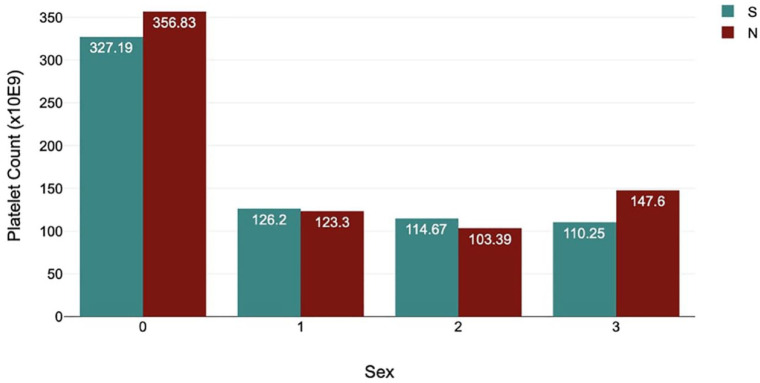
Bar graph comparing sex, mean platelet count, and degree of observable platelet clumping in cats included in a study of platelet characteristics. N = neutered males; S = spayed females

No significant association was found between sex and platelet clumping, regardless of whether intact animals were included (*P* = 0.323) or excluded (*P* = 0.269) (Figure 1).

**Figure 2:**
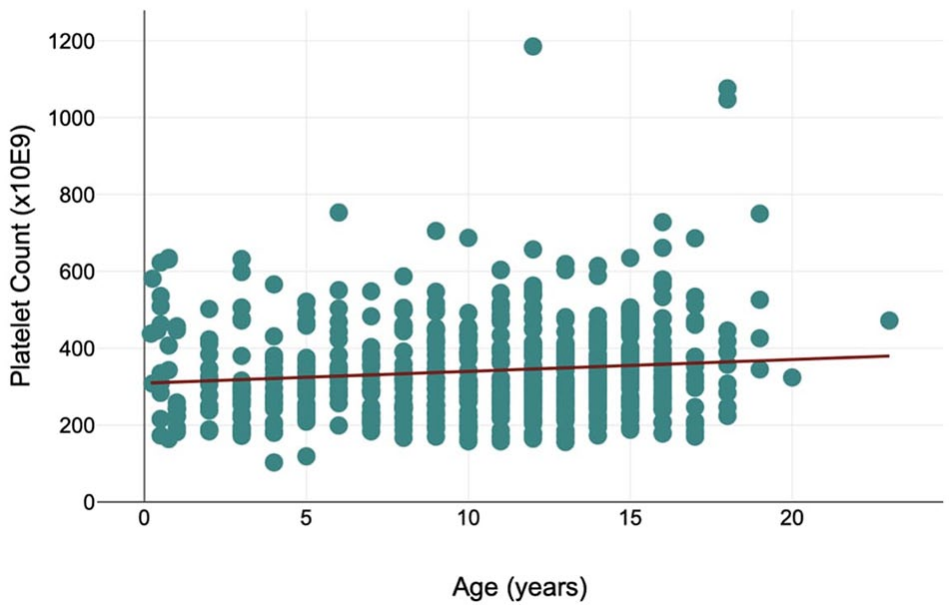
Scatter diagram showing the platelet counts of cats with no observable platelet clumping, included in a study of platelet characteristics by age (range 2 months to 23 years)

Age was not associated with platelet clumping (*P* = 0.959). A significant (*P* = 0.003) positive correlation was present between age and platelet count, with an increase in platelet count of 2.14 × 10^9^/l (95% confidence interval = 0.21–4.49 × 10^9^/l) for each year of age (Figure 2).

**Figure 3:**
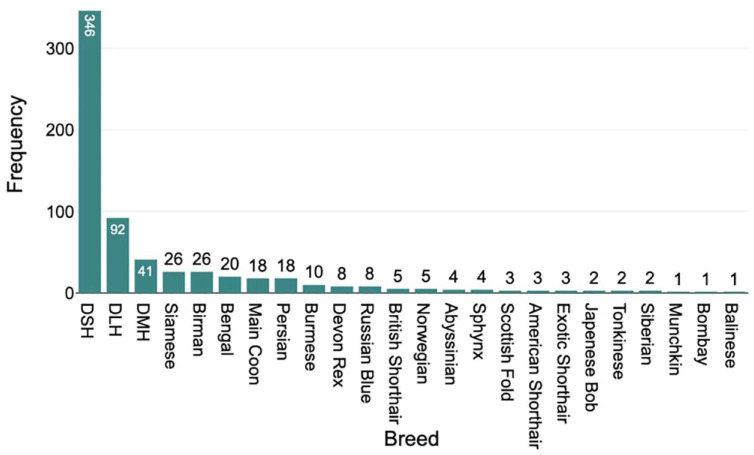
Histogram showing the relative frequency of breeds of cat included in a study of platelet characteristics. DLH = domestic longhair; DMH = domestic mediumhair; DSH = domestic shorthair

The majority of the study population were domestic cats. However, a variety of other breeds were included (Figure 3). After dichotomization, the domestic group (domestic shorthair, domestic medium hair and domestic longhair) contained 418 cats and the purebred group contained 161 cats. There was no statistically significant effect of purebred status on platelet count (*P* = 0.457). The domestic group had a significantly higher rate of platelet clumping than the purebred group (*P* = 0.009).

**Figure 4:**
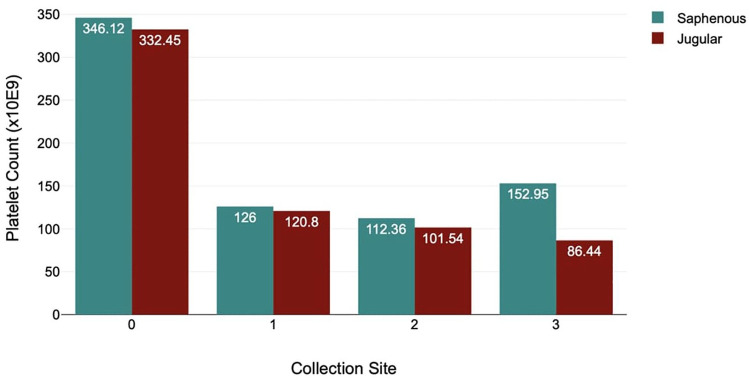
Bar graph comparing collection site, mean platelet count, and degree of observable platelet clumping (see Table 1) in cats included in a study of platelet characteristics

No association was found between platelet count and collection site (saphenous 315 × 10^9^/l, jugular 313.5 × 10^9^/l; *P* = 0.322). Platelet clumping was also not associated with collection site (*P* = 0.793) (Figure 4).

When platelet clumping was considered as a binary outcome, there was no association between clumping and sex (*P* = 0.292), age (*P* = 0.681), site of collection (*P* = 0.809) or breed (*P* = 0.264).

Platelet morphology was normal in 637 of the samples, with shift platelets (larger than red blood cells) found in 12 cats. The presence of shift platelets was positively associated with age (*P* <0.01) and with the presence of thrombocytosis (*P* <0.001).

## Discussion

This study investigated the association of breed, sex, age and site of collection with platelet count and degree of platelet clumping. Female cats were found to have lower platelet counts than males, in alignment with previous studies in cats.^8^ This is contrary to the situation in humans, where women are well established to have a higher platelet count than men.^9,11^ In dogs, research is inconclusive; de Marcos Carpio et al.^19^ did not find a difference in platelet count based on sex, whereas Bourgès-Abella et al.^25^ found that females had a higher platelet count.

Explanations for the differences between humans and cats include hormonal causes not seen in neutered pets, and differences in body composition that may be less present in cats.^12,13,26^ In human medicine, women may require higher doses of anti-platelet drugs.^3,27,28^ Female cats have documented higher concentrations of the active metabolite of clopidogrel, and this is suspected to be due to tissue composition and relative differences in tissue solubility as opposed to sex-related differences in clopidogrel metabolism.^29^ The findings of this study support previous research suggesting that sex may also play a role in antithrombotic therapy in feline medicine.^29^ Considering the sample size of intact females (n = 1) vs spayed females (n = 279), these changes are unlikely to be due to sex hormones. More research is needed to confirm the findings in this study and to understand the basis behind the lower platelet counts seen in female cats.

The significant but small positive correlation between age and platelet count was also contrary to the findings in humans, where age is negatively associated with platelet count.^9,10,30^ In a previous study in cats,^8^ a negative association between age and thrombocytopenia was found on univariable but not multivariable analysis. No other data on age and platelet count are published on cats; however, in a variety of different canine breeds, no correlation between platelet count and age was found.^19^

The occurrence of thrombocytosis and thrombocytopenia in this study is similar to previously reported data.^4,6,7^ Therefore, as thrombocytosis is often associated with neoplasia or inflammatory disease in veterinary medicine, its association with age is not surprising.

This study did not find a difference in platelet count between domestic and purebred cats, consistent with previous data.^8^ In dogs, breed differences in platelet count have not generally been reported, with the exception of macrothrombocytopenic breeds.^19^ However, platelet count in humans has been documented to vary based on ethnicity.^10^ This study suggests that domestic cats have higher rates of platelet clumping than purebred cats. Possible reasons for this finding may include differences in platelet reactivity, patient temperament or disease states based on breed.

The lack of association of collection site/technique with platelet count is of significant clinical relevance. These results align with the findings of previous studies^22,23^ that found no difference in platelet count between blood samples collected by direct jugular venipuncture and a vascular access port or jugular catheter, and with another study^24^ that did not find a difference in platelet count when 25 G vs 22 G needles were used. Studies in dogs agree with these findings, suggesting that neither collection site nor needle gauge had a meaningful effect on platelet count.^31,32^ This suggests that, although the traditional approach in studies on feline platelets has been jugular venipuncture,^33–35^ multiple sites of collection are likely reasonable.

The occurrence of pseudothrombocytopenia in cats is likely related to a combination of factors including the size of platelets and their tendency to clump during handling and collection.^36^ Feline platelets are a similar size to erythrocytes and are larger than those of dogs, pigs or humans, and may be falsely counted as erythrocytes by some automated analyzers.^5^ The light-scattering pattern of a platelet clump is different than that of a single platelet, and so some optical counters will exclude aggregates.^5^ Challenges continue to exist, even with new analyzers such as the XN-V analyzer (Sysmex), which is capable of partially identifying platelet aggregates and sends a ‘platelet clumps?’ flag.^37^ This analyzer is not able to identify all aggregates and additional studies are needed to understand its accuracy in determining true vs pseudothrombocytopenia.^37^ Blood film review in light of a low platelet count is still recommended despite technological advances.^37^

This study has several limitations. These include the small numbers of purebred cats, unknown body weights and non-randomized blood collection site selection. Time to the laboratory and transportation factors may have also altered platelet counts recorded on the XT-V analyzer (Sysmex). Time has been documented to effect platelet counts^4^ but recent research has revealed that it has limited effect on platelet function tests.^38,39^ Since counts were run only in the laboratory and not on an in-hospital machine, it is challenging to comment on this effect. The study did not account for or record underlying diseases and reasons for blood collection; however, it did exclude cats if they were receiving clopidogrel or aspirin or if they had a disease known to affect platelet function. Mean platelet volume and plateletcrit were not reported by the analyzer used in this study and, as such, are not available for analysis.

A study with larger sample sizes of a variety of different pure breeds may be able to find significant differences in platelet count and clumping that this study could not, possibly gaining more information on the finding that domestic cats’ platelets clumped more than those of purebred cats. Considering genetic differences exist in cats regarding their platelets, breed differences in feline platelets warrant more research.^20^

The effect of weight and body condition score on platelet count would have been interesting to analyze. Unfortunately, body weights were not recorded, and so this effect could not be explored. This limits our ability to determine whether increased body mass index increases platelet counts in cats, as it does in humans.^12,13^

The collection site was not randomized, and so confounding factors such as patient temperament may have influenced the choice of collection site. The records did not note the difficulty of collection, which could play a role in clumping. There is also the need to consider that different phlebotomists may have preferred certain veins, and so this may be a confounding variable. Only two possible collection methods were assessed, and others, such as via a catheter, different needle gauges, syringe sizes, and so on, were not assessed. However, this number of variables would have been impractical for a single-center trial. All blood samples were performed at a feline specialty hospital by experienced phlebotomists and as such these results may be applicable only to these circumstances.

## Conclusions

This study demonstrated a statistically (although potentially not clinically) relevant sex difference in platelet count in cats, with females having lower platelet counts than males. It also identified a trend toward higher platelet counts in older cats and increased platelet clumping in domestic compared with purebred cats. Most significantly, it identified no difference in platelet count or degree of platelet clumping based on collection site. It also found that platelet clumping was not associated with sex or age in cats. This understanding of the effects of signalment on feline platelet variability may be useful for interpretation of platelet counts and platelet function testing, and in monitoring response to therapeutics. Further research to confirm and expand on these findings should include larger numbers of purebred cats, differing methods of collection and assessment of different health conditions.
